# The Mongoose, the Pheasant, the Pox, and the Retrovirus

**DOI:** 10.1371/journal.pbio.1001641

**Published:** 2013-08-27

**Authors:** Lucie Etienne, Michael Emerman

**Affiliations:** Division of Human Biology, Fred Hutchinson Cancer Research Center, Seattle, Washington, United States of America

## Abstract

Paleovirology is the study of ancient viruses. The existence of a paleovirus can sometimes be detected by virtue of its accidental insertion into the germline of different animal species, which allows one to date when the virus actually existed. However, the ancient and the modern often connect, as modern viruses have unexpected origins that can be traced to ancient infections. The genomes of two species of mongooses and an egg-laying mammal called an echidna show that a virus currently present in poultry, the reticuloendotheliosis virus (REV), is actually of ancient exotic mammalian origin. REV apparently spread to poultry through a circuitous route involving the isolation of malaria parasites from a pheasant from Borneo housed at the Bronx Zoo that was contaminated with REV. Repeated passage of this virus in poultry adapted the virus to its new host. At some point, the virus got inserted into another virus, called fowlpox virus, which has spread back into the wild. Although REV may still exist somewhere in a mammalian host, its modern form links an 8 million-year-old infection of the ancestor of a mongoose to a virus that now is circulating in wild birds through malaria studies in the mid-20^th^ century. These lessons of ancient and modern viruses have implications for modern human pandemics from viral reservoirs and for human interventions that may come with unintended consequences.

Outbreaks of “new” viruses are real causes of concern for human and animal health. But where do they come from? The vast majority of emerging viruses in the human population originate from other animals [1]. In fact, when a new virus appears, it is often a virus that already exists in one species, but then becomes established in another. For example, the transmission of a coronavirus (CoV) from bats to palm civets and raccoon dogs and ultimately to humans led to the emergence of severe acute respiratory syndrome (SARS-CoV), which created an epidemic in 2003, with 8,000 people infected and 700 deaths before it was finally eliminated in humans [2]. Another example is the transmission of influenza virus from birds to mammals (pigs and humans) in 1918 that was responsible for over 100 million deaths worldwide [3], and many of the genes from this virus continue to circulate in human influenza to this day.

Whether or not a virus will “emerge” in new species depends on a complex set of requirements including contact, mode of transmission, and adaptation to the new species [4],[5]. Moreover, human interventions can sometimes accidentally facilitate the transfer of infectious agents to an animal species where they did not previously exist. For example, the spread of “mad cow” disease from sheep to cattle (and then subsequently to humans) in the 1980s occurred through changes in how cattle feed was prepared [6]. In about the same time-period, the spread of a relative of HIV, the simian immunodeficiency virus (SIV), from an African monkey species to a previously uninfected Asian monkey, took place in primate centers in the United States [7]. Even more dramatically, the epidemic appearance of seemingly “new” viruses can be unwittingly aided by human activities that amplify previously rare viruses, as was seen with the reuse of hypodermic needles in campaigns against schistosomiasis (snail fever) that led to the exponential spread of hepatitis C virus in Egypt in the mid-20th century [8], as well as similar, but more speculative, theories for the amplification of HIV in central Africa during campaigns to eliminate *Trypanosoma brucei* (sleeping sickness) in the 1920s [9].

Ultimately, though, where did these viruses come from? Fossils of animals and plants have been used to understand the age of groups of species. Although no physical fossils exist for viruses, clues about virus history can be gleaned from the remnants of viral sequences (called “viral fossils”) that have been accidently inherited in the genome of the host they infected in the past. That is, although viruses typically spread between hosts by transmission from one individual to another (called horizontal transmission), on rare occasions viral nucleic acids can get integrated into the host germline and become endogenous viral elements (EVEs) [10]. These EVEs can then be transmitted from parent to offspring (called vertical transmission) by inheritance of chromosomes that contain the viral DNA. At some rare frequency, these inherited EVEs become present in all members of a species (called “fixation”) and will evolve along with the host genome over extended periods of time (Figure 1). The analyses of the host genomic data can thus reveal the presence of these EVEs, which represent “viral fossil records” and can provide information on ancient viruses; together these analyses are known as “direct” paleovirology (in contrast to “indirect” paleovirology, which instead looks at the consequences of ancient viruses on the evolution of host genes) [11]. Determination of the presence or absence of shared EVEs in different species can be used to estimate the date of the viral insertion into the host genome [10] and to infer the presence of an ancient circulating virus among certain hosts over a very long period of time (Figure 1). For example, the analyses of EVEs among different species showed that a relative of the hepatitis B virus that currently infects humans was present in the passerine birds at least 19 million years ago [12]. Importantly, because integration in the host cell chromosome is an obligate part of the lifecycle of retroviruses, the catalog of endogenous retroviral elements provides an especially rich record of past retroviral infections (for example, [13]).

**Figure 1:**
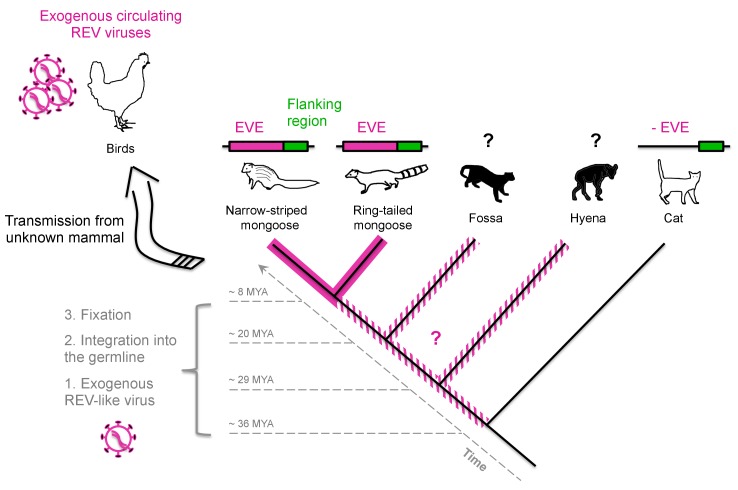
Paleovirology: How the study of endogenous viral elements (EVEs) informs us about the evolutionary history of modern viruses. The concept is illustrated with the example of the viral fossil record and evolutionary history of the reticuloendotheliosis virus (REV). The tree is a timescaled representation of a host genome phylogeny. Hosts for which genomic sequences are available are depicted in white, while hosts that do not have genome data available are represented in plain black. The hosts that harbor a REV-like endogenous element are depicted, as well as those from which information is not available (question marks) and others from which the EVE is absent. Regions in green represent the EVE flanking region. The time frame in which an exogenous mammalian virus (1), here REV, integrated in the host germline (2) and reached fixation (3) is represented. The presence of related EVEs at the same locus in both mongoose species shows that the insertion occurred before their divergence, approximately 8 million years ago (MYA), while the absence of EVE at this site in the cat species indicates that the insertion occurred after 36 MYA. Although not definitive, PCR for REV in fossa was negative [21]. Here, a more recent inter-class transmission from an unknown mammal was the origin of REV in birds. Image Credit: Lucie Etienne.

Among retroviruses, a group of viruses related to the reticuloendotheliosis virus (REV) has especially interesting ancestral origins. REV currently circulates in chickens, turkeys, pheasants, ducks, and geese, where it sometimes causes disease. However, its genome is not related to other avian retroviruses; rather, it is most closely related to some mammalian retroviruses [14]. This is unusual because retroviral transmissions between different classes of animals (e.g., between birds and mammals), called inter-class transmissions, are much more difficult than those between closely related species and are often dead ends [15]. Indeed, the virus needs to adapt to large differences between the host species that will include, among many other things, differences in the host cell receptors to allow the virus to enter the cells, differences in the host cell environment (e.g., temperature) that the virus needs to replicate, and different sets of immune defense proteins. One hypothesis is that REV may have been aided in its ability to move between very different species because it is a an unusual recombinant virus between two different retrovirus genera; some of the REV genes are related to gamma-retroviruses, while the viral gene that encodes the protein that allows it to enter cells is derived from beta-retroviruses [14]. There are several important biological differences between gamma-retroviruses and beta-retroviruses, but one that may be important in understanding the origins of REV is that the gamma-retroviruses do not generally undergo inter-class transmissions, while the fossil records of beta-retroviruses show more propensity for large species jumps [16]–[18]. Finally, REV is unusual in having integrated itself into two other large DNA viruses: a herpesvirus, which replicates in the nucleus, and a poxvirus, which replicates in the cytoplasm [19],[20].

In this issue of *PLOS Biology*, Niewiadomska and Gifford elucidate the complex evolutionary history of REV and related viral strains by using paleovirology and a historical review of the literature to infer the viral origin, the successive inter-species transmissions, and the adaptive events that led to the circulation of REVs in wild birds [21]. Along the way, the story takes us from a mongoose, to a pheasant at the Bronx Zoo, to a duck model of malaria that might have led to contamination of vaccines of large DNA viruses of birds, which may have subsequently spread REV around the world.

The link between this avian retrovirus and endogenous elements in mammals was first documented in a screen of available animal genomes in which the most closely related sequence to REV was from a fragment of the genome of an echidna, an egg-laying mammal from Australasia [14]. Niewiadomska and Gifford were characterizing the diversity of endogenous retroviral elements in Malagasy carnivores using previously frozen tissue samples, and fortuitously happened upon REV-like sequences in the genome of the ring-tailed mongoose and the narrow-striped mongoose [21]. Additional DNA sequencing identified viral sequences that were similar to REV across the entire viral genome, including the same recombination breakpoint found in modern REV. This crucial finding showed definitively that the endogenous retrovirus from mammals and the currently circulating avian exogenous REVs share a common ancestor. To estimate the age of this ancient retroviral lineage, the authors showed that the flanking regions of the newly characterized EVEs were common to the two mongoose species [21], thus showing the REV-like retrovirus entered the germline and reached fixation before the split between these two species during the Miocene period (Figure 1). Therefore, an exogenous recombinant REV-like virus was circulating in mammals more than 8 million years ago.

How then did this mammalian retrovirus get into birds? From here the story gets spectacular and strange (yet still very plausible). In the 1930s, the need for an experimental model system for malaria research led Lowell Coggeshall to isolate *Plasmodium* parasites from a pheasant, originally from Borneo, that was housed at the Bronx Zoo. For experimental vaccine and drug research purposes, the *Plasmodium* stock was passaged over time in poultry until the 1980s and was distributed to many US laboratories. However, the culture of *Plasmodium lophurae* was contaminated with retroviruses from the REV lineages (contamination may have existed in the pheasant, or might have been acquired during passage). The authors depict a compelling scenario in which the zoological park, an environment in which exotic animal species from diverse lineages are brought together in unusually close proximity, could have favored the inter-class transmission of REVs from mammals to the pheasant from which *Plasmodium* was isolated. However, as inter-class transmissions often result in dead-end infections, the authors surmise that serial passages of the retrovirus-containing *Plasmodium* stock in poultry favored the adaptation of the mammalian virus to bird hosts. Sometime along the way, the REV virus integrated into two large DNA viruses: a poxvirus (FWPV) that contained a full-length insertion of REV and a herpesvirus (GHV-2) containing only a fragment of REV [19],[20]. Because live attenuated FWPV vaccines harboring replicative competent REVs were used in vaccination campaigns of poultry, it may have further played a role in the spread of REVs into wild birds around the world [21]. Thus, REV is now a bird virus that was once an ancient mammalian virus (Box 1).

**Box 1. Scientific Advances Using the Reticuloendotheliosis Viruses**

While the main text describes the origins of the reticuloendotheliosis virus (REV) and its close relatives as pathogens, the study of these retroviruses has also contributed to several areas of basic science and basic virology. The original description of REV was from a tumor taken from a turkey that contained two viruses. One, REV-A, was replication-competent, and the other, REV-T, was defective, but carried an oncogene called Rel that allowed it to transform cells [23],[24]. Rel, it turns out, is a key component in the innate immune signaling pathway [25], as a component of the NF-κB transcription factor [26]. The late Howard Temin adopted REV and related viruses as model systems to describe, among many other phenomena, the kinetics of retroviral infections [27], the derivation of cell lines for making gene therapy vectors [28], and retrovirus mutation and recombination rates [29],[30].

Further efforts are needed to determine if REV still has a modern mammalian host; the authors of this study speculate it could be a bat species [21]. Indeed, the reservoir of viruses is vast, and recent field studies and metagenomic screens are finding relatives of human and domestic animal viral pathogens in unexpected places (for example, [22]). Moreover, it will be important to determine if the ancient exogenous REV had a wider reservoir by investigating more animal genomes (Figure 1). Importantly, the increasing number of full-length genome data obtained by next-generation sequencing from various animal species will reveal an even greater number and genetic diversity of EVEs, which will allow us to understand better the ancestors of the modern viruses and estimate with greater precision the date of their viral insertion into the host germline (Figure 1). Thus, the answer to the question “where did these viruses come from?” will increasingly become “they came from ancient forms of other viruses,” although in some cases, such as those described for REV [21], they come through a thoroughly circuitous route.

## Abbreviations

CoV: coronavirus
EVE: endogenous viral element
MYA: million years ago
SARS: severe acute respiratory syndrome
REV: reticuloendotheliosis virus
SIV: simian immunodeficiency virus
